# Gonioscopy-assisted transluminal trabeculotomy (GATT) outcomes in eyes with open-angle glaucoma resistant to maximum treatment

**DOI:** 10.5935/0004-2749.20210083

**Published:** 2025-08-21

**Authors:** Bruno M. de Faria, Fábio B. Daga, Vespasiano Rebouças-Santos, Rafael B. de Araújo, Carlos Matos Neto, Jéssica S. Jacobina, Marco A. R. de Faria

**Affiliations:** 1 Department of Ophthalmology, School of Medicine, Universidade Federal do Rio Grande do Norte, Natal, RN, Brazil; 2 Department of Ophthalmology and Vision Science, Escola Paulista de Medicina, Universidade Federal de São Paulo, SP, Brazil; 3 Ver Excelência em Oftalmologia, Goiânia, GO, Brazil; 4 Hospital Santa Luzia, Salvador, BA, Brazil

**Keywords:** Trabeculectomy/methods, Glaucoma, open-angle/surgery, Gonioscopy/methods, Treatment outcome, Trabeculotomia/métdos, Glaucoma de ângulo aberto/cirurgia, Gonioscopia/métodos, Resultado de tratamento

## Abstract

**Purpose:**

To report the initial 2 years’ learning curve on gonioscopy-assisted transluminal trabeculotomy performed using the thermally blunted suture technique and review the factors that could potentially affect the outcome.

**Methods:**

This retrospective study evaluated 100 eyes from 89 participants with glaucoma resistant to maximum clinical treatment, which was defined as having an intraocular pressure >21 mmHg in addition to three or four different hypotensive drugs. Intraocular pressure values at baseline, 1 week, and at 1, 2, 3, 6, 12, and 24 months of follow-up and details regarding the need of antiglaucoma medication and further glaucoma surgery were recorded. Eyes that required further surgical intervention for intraocular pressure control were considered as failure.

**Results:**

A total of 51 eyes were subjected to isolated gonioscopy-assisted transluminal trabeculotomy, and 49 eyes were subjected to gonioscopy-assisted transluminal trabeculotomy + cataract extraction at the same surgical time. A statistically significant difference was observed between overall mean follow-up intraocular pressure and mean preoperative intraocular pressure (p<0.001) in all follow-up visits. When the extent of treatment was evaluated, patients with an extension of 360° did not exhibit statistically significantly lower mean intraocular pressure than those with other extensions. Hyphema was the only complication presented in 50 eyes (50%), but all had spontaneous resolution within 4 weeks. A total of 26 eyes (26%) required additional conventional trabeculectomy due to uncontrolled intraocular pressure, especially those who previously underwent vitreoretinal surgery.

**Conclusions:**

Gonioscopy-assisted transluminal trabeculotomy, besides being an apparently safe procedure, results in satisfactory success rates even during the surgeon’s initial learning curve. The technique was effective in decreasing intraocular pressure and medication burden.

## Abstract (pt)

**Objetivo:**

Reportar a curva de aprendizado dos 2 anos iniciais da trabeculotomia transluminal assistida por gonioscopia, usando a técnica de sutura termicamente atenuada e revisar os fatores que podem afetar o resultado.

**Métodos:**

Este estudo retrospectivo incluiu 100 olhos de 89 participantes com glaucoma resistente ao tratamento clínico máximo, definido como tendo pressão intraocular superior a 21mmHg, além de três ou quatro drogas hipotensoras diferentes. Pressão intraocular inicial, 1 semana, primeiro, segundo, terceiro, sexto, 12 e 24 meses de acompanhamento; necessidade de medicação antiglaucoma; necessidade de mais cirurgias anti-glaucomatosas foram registradas. Olhos que necessitaram de intervenção cirúrgica adicional para o controle da pressão intraocular foram considerados como insucesso.

**Resultados:**

Cinquenta e um olhos foram submetidos à trabeculotomia transluminal assistida por gonioscopia isolado e 49 olhos à trabeculotomia transluminal assistida por gonioscopia associado à extração de catarata no mesmo tempo cirúrgico. Houve diferença estatisticamente significativa entre a pressão intraocular média global no acompanhamento e a pressão intraocular média pré-operatória (p<0,001) em todas as visitas do acompanhamento. Ao avaliar a extensão do tratamento, os pacientes com extensão de 360 graus não apresentaram pressão intraocular **média menor** estatisticamente significativa em comparação com outras extensões. O hifema foi a única complicação presente em 50 olhos (50%), contudo todos tiveram resolução espontânea em quatro semanas. Um total de 26 olhos (26%) teve que ser submetido a trabeculectomia convencional adicional devido à pressão intraocular descontrolada, principalmente aqueles previamente submetidos à cirurgia vitreorretiniana.

**Conclusões:**

A trabeculotomia transluminal assistida por gonioscopia, além de ser um procedimento aparentemente seguro, apresenta taxas de sucesso satisfatórias, mesmo durante a curva de aprendizado inicial do cirurgião. A técnica foi efetiva em reduzir a pressão intraocular e uso de medicamentos.

## INTRODUCTION

Lowering intraocular pressure (IOP) is the common goal of all of the different glaucoma treatment strategies. Among surgical strategies, trabeculectomy has been the most widely and successfully performed procedure for decades. In around 70% of cases, IOP was appropriately controlled after the procedure^(1)^. However, this surgical technique has some risks and complications such as hypotony, aqueous leakage, bleb-related infections, choroidal detachment, and induced astigmatism^(2)^.

On the other hand, the *ab externo* trabeculotomy procedure, another technique for lowering IOP, which consists of removing the trabecular meshwork, the first mechanical barrier for aqueous humor outflow, improves drainage through Schlemm’s canal and collector channels, with the advantage of avoiding the need for bleb construction^(3)^. For more than 50 years, several authors have demonstrated the efficacy of the *ab externo* trabeculotomy procedure in lowering IOP, especially in congenital and juvenile glaucoma^(3-7)^.

In recent years, there has been a global increasing interest and development of several minimally invasive glaucoma surgery (MIGS) techniques, especially because of its safer surgical profile. More recently, Grover et al. described a pioneering technique of *ab interno* trabeculotomy termed as gonioscopy-assisted transluminal trabeculotomy (GATT)^(8)^. They reported good efficacy in reducing IOP, in addition to excellent safety results in a short-term evaluation, using both an optic fiber microcatheter (iTrack^®^, Ellex, California) and a thermally blunted suture^(9)^. Current research also demonstrated that in addition to significantly reducing the IOP and number of medications, it had fewer postoperative complications^(10,11)^. GATT may even be an effective and safe option for patients with refractory glaucoma who have undergone a previous incisional surgery^(12)^. Furthermore, it is significantly more affordable to perform GATT procedure using the thermally blunted suture than using the microcatheter.

Till date, there have been only a few studies that have evaluated the reproducibility of the thermally blunted suture technique^(8,9,13,14)^. Moreover, no previous study has yet evaluated the possible outcomes using different *ab interno* trabeculotomy extensions (i.e., 180° or 360°). Therefore, the aims of this study were to report our 2 years of follow-up results obtained using the thermally blunted suture GATT technique performed in patients with open-angle glaucoma (OAG) resistant to clinical treatment and to review factors such as the treatment extension that could potentially affect the outcome.

## METHODS

This retrospective study evaluated the data of participants who were subjected to GATT procedure during March 2017 and August 2019 at the following institutions: *Hospital Universitário Onofre Lopes*, Natal, Brazil, and *Hospital Santa Luzia*, Salvador, Brazil. All subjects were operated by two surgeons, and the results described in this study represent their learning curve experience. The institutional review board and the human subjects committee approved all methods. All study methods adhered to the tenets of the Declaration of Helsinki for research involving human subjects, and the study was conducted according to the regulations of the Health Insurance Portability and Accountability Act.

Only subjects with open angles on gonioscopy, besides easy identification of angle structures and landmarks, were included in this study. Subjects were excluded if they presented any signs of anterior chamber neovascularization, anterior peripheral synechiae, or any other angle abnormalities. Subjects diagnosed with topical resistant glaucoma, defined as having an IOP >21 mmHg besides maximum treatment (three or four different hypotensive drugs), were included in this study. When both eyes of subjects had an IOP >21 mmHg besides maximum treatment, both eyes were included in the study. No other eyes were excluded for any other reason. Some participants underwent both cataract surgery and GATT procedure at the same time, when indicated. Eyes that required further surgical intervention for IOP control at any time during the follow-up were considered as failure.

This study was conducted without patient involvement. Patients were not invited to comment on the study design and were not consulted to develop patient-relevant outcomes or interpret the results. Patients were not invited to contribute to the writing or editing of this document for readability or accuracy.

### GATT Procedure

**Figure 1:**
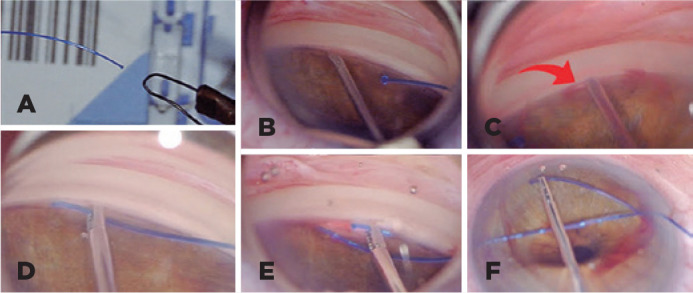
A) Photograph demonstrating a low-temperature ophthalmic cautery used to blunt the tip. B) A goniotomy is created with a microsurgical blade. C) Use of viscoelastic in order to confirm the Schlemm’s canal openin . D) Microsurgical forceps are used to cannulate the Schlemm canal through the goniotomy site and pass the polypropylene suture. D and E) The suture is retrieved through the goniotomy site with traction being placed on the distal and proximal portion of the suture, and the goniotomy is performed.

The complete surgical technique has been previously described^(8)^. In brief, after superior nasal and temporal corneal paracentesis, a solution of xylocaine and myostat was injected in the anterior chamber. Then, the anterior chamber was filled with a viscoelastic material. Using the tip of a 26-gauge needle, a nasal goniotomy was created. A thermally blunted 5-0 polypropylene suture was then inserted through the goniotomy and circumferentially advanced using the serrated tip of 23-gauge microsurgical forceps (Figure 1). The distal tip of the suture was advanced 360°, retrieved at the nasal goniotomy site, and extracted from the anterior chamber, thus creating circumferential trabeculotomy. In some cases where anatomical resistance was detected while advancing the suture, a new goniotomy was created to retrieve the distal tip, creating a circumferential trabeculotomy that varied from 90° to up to 360°. The viscoelastic was then removed from the anterior chamber by anterior chamber irrigation with a basic saline solution. Acute hypotony and hyphema were controlled by anterior chamber viscoelastic injection. The amount of viscoelastic left in the eye was based on the degree of blood reflux as well as the presence and degree of episcleral venous fluid wave^(15)^.

For cases in which both cataract extraction (CE) and GATT procedure were performed, phacoemulsification with intraocular lens implantation was performed initially, and then the trabecular meshwork was accessed with nasal goniotomy following the *ab interno* trabeculotomy. The same paracentesis was used for goniotomy and phacoemulsification, with no difference from the ordinary phacoemulsification surgical technique.

Subjects were followed up with broad-spectrum antibiotics for 7 days and tapering topical steroids for 1 month. In addition, pilocarpine (2%) was used BID for 7 days, followed by once a day for 7 days. All cases were evaluated preoperatively and followed up at days 1 and 7 and months 1, 2, 3, 6, 12, and 24. Reintroduction of topical drugs for IOP control was also evaluated.

### Statistical analysis

Descriptive statistics consisted of mean and standard deviation of variables. Normality assumption was evaluated by examination of histograms and using the Shapiro-Wilk test. Wilcoxon paired tests were used to compare preoperative and postoperative measurements. Comparisons between subgroups were performed using Mann-Whitney and Kruskal-Wallis tests. Kaplan-Meier survival curves were used to evaluate failure rates.

All statistical analyses were conducted using the commercially available software Stata, version 14 (StataCorp LP, College Station, TX). The alpha level (type I error) was set at 0.05.

## RESULTS

This study included 100 eyes from 89 participants. table 1 shows the demographic and clinical characteristics of the study population. The age of the participants ranged from 6 to 84 years, with the mean age being 59.18 ± 15.54 years. Mean preoperative IOP was 24.85 ± 9.00 mmHg. A total of 51 eyes were subjected to isolated GATT procedure, and 49 eyes were subjected to the GATT procedure with phacoemulsification at the same surgical time. The types of glaucoma that were included in this study are presented in table 2.

**Table 1:** Demographics and clinical characteristics of participants included in the study

Eyes (participants)	100 (89)
Mean age (Range)	59.18 ± 15.54 (6-84)
Mean pre-op IOP (Range)	24.85 ± 9.00 (11-52)
Female sex, n (%)	34 (34.0)
Isolated GATT, n (%)	51 (51.0)
GATT + CE, n (%)	49 (49.0)

IOP= intraocular pressure; GATT= gonioscopy-assisted transluminal trabeculotomy; CE= cataract extraction.

Values are presented as mean ± standard deviation, unless otherwise noted.

**Table 2:** Types of glaucoma included in the study

POAG	69 (69.70)
Secondary to vitreoretinal surgery	12 (12.12)
Steroid-induced glaucoma	10 (10.10)
Congenital glaucoma	5 (4.04)
Pigmentary glaucoma	2 (2.01)
Uveitic glaucoma	1 (1.01)
Glaucoma after trauma	1 (1.01)

POAG= primary open-angle glaucoma.

Values are presented as total number and percentage of total (%).

Some participants were lost during the follow-up period, especially at the 1- and 2-year visits. In the isolated GATT group, 18 participants were lost from 6 months onward, and an additional 14 and 4 participants were respectively lost at the 1- and 2-year visits. In the CE + GATT group, 12 participants were lost at 6 months, and an additional 13 participants were each lost at the 1- and 2-year visits, respectively. Mean IOP values measured during the follow-up at day 7 and months 1, 2, 3, 6, 12, and 24 were respectively 14.30 ± 7.25, 14.66 ± 6.70, 13.79 ± 5.38, 12.53 ± 3.12, 12.81 ± 3.12, 12.63 ± 2.01, and 12.58 ± 1.24 mmHg. The overall mean follow-up IOP and mean preoperative IOP were statistically significantly different (p<0.001) in all follow-up visits (Table 3). Furthermore, when the groups were evaluated separately (isolated GATT and GATT + CE), the overall mean follow-up IOP was statistically significantly different from preoperative IOP values (p<0.001).

**Table 3:** Outcome data for all eyes over the course of the study

		All eyes	Isolated GATT group	GATT + CE group	
Patients at follow-up visits n (%) Preoperative		100 (100.00)	51 (100.00)	49 (100.00)	
7 days		84 (84.00)	41 (80.39)	43 (87.76)	
One month		90 (90.00)	45 (88.24)	45 (91.84)	
Two months		84 (84.00)	41 (80.39)	43 (87.76)	
Three months		68 (68.00)	33 (64.71)	35 (71.43)	
Six months		70 (70.00)	33 (64.71)	37 (75.51)	
One year		43 (43.00)	19 (37.25)	24 (48.98)	
Two years		26 (26.00)	15 (29.41)	11 (63.64)	
	Mean IOP (mmHg)				
	Preoperative	24.85 ± 9.00	26.25 ± 8.34	23.39 ± 9.51	
	7 days	14.30 ± 7.25	14.78 ± 7.81	13.84 ± 6.74	
	One month	14.66 ± 6.70	16.07 ± 7.55	13.24 ± 5.46	
	Two months	13.79 ± 5.38	15.02 ± 6.08	12.60 ± 4.36	
	Three months	12.53 ± 3.12	13.09 ± 3.54	12.00 ± 2.61	
	Six months	12.81 ± 3.12	13.42 ± 3.83	12.27 ± 2.23	
	One year	12.63 ± 2.01	13.11 ± 2.49	12.25 ± 1.48	
	Two years	12.58 ± 1.24	12.87 ± 1.30	12.18 ± 1.08	
Mean no. of medications (SD) Preoperative		3.61 ± 0.75	3.90 ± 0.61	3.31 ± 0.77	
7 days		0.83 ± 0.99	0.95 ± 1.08	0.72 ± 0.88	
One month		1.03 ± 1.18	1.16 ± 1.19	0.91 ± 1.16	
Two months		1.21 ± 1.38	1.54 ± 1.43	0.91 ± 1.27	
Three months		1.04 ± 1.33	1.33 ± 1.47	0.77 ± 1.14	
Six months		1.12 ± 1.31	1.30 ± 1.38	0.94 ± 1.24	
One year		1.51 ± 1.30	1.47 ± 1.26	1.54 ± 1.35	
Two years		0.96 ± 1.04	1.07 ± 1.16	0.82 ± 0.87	

GATT= gonioscopy-assisted transluminal trabeculotomy; CE= cataract extraction; SD= standard deviation; IOP= intraocular pressure. All follow-up measurements were significantly different from preoperative values.

Values are presented as mean ± standard deviation, unless otherwise noted.

* Statistical significance evaluated by Chi-square test.

There was no statistical difference in the number of medications postoperatively between the groups in any of the visits (p>0.05 for all). However, there was a reduction in the number of medications from the preoperative period to the day 7 visit and all the other follow-up visits (p<0.05 for all) (Table 3).

Hyphema was the only complication during the initial postoperative period, with 50 eyes (50%) developing hyphema. However, all cases showed spontaneous resolution within 4 weeks, with a mean resolution time of 4.7 ± 6.9 days. The other complications previously reported with GATT, such as Descemet’s membrane detachment, corneal edema, iridodialysis, and cystoid macular edema, were not observed in this population^(8)^.

A total of 26 eyes (26%) required additional surgery, which included 14 patients with primary OAG, 8 patients with glaucoma secondary to vitreoretinal surgery, 4 patients with congenital glaucoma, 1 patient with glaucoma after trauma, and 1 patient with pigmentary glaucoma. A total of 12 patients underwent trabeculectomy (46.2%), 8 patients received micropulse treatment (30.8%), and 6 patients received implant devices (23.1%). There was no significant relationship between GATT failure and presence of hyphema (p=0.192). Moreover, no relationship was observed between preoperative IOP measurements, number of quadrants treated, number of medications in the preoperative period, association with CE, age, or gender (p>0.05 for all).

We also evaluated whether the extent of treatment, i.e., the number of quadrants where GATT was performed, had an impact on IOP levels. GATT to 360° (four quadrants) was performed on 77 eyes (77%), to 270° (three quadrants) was performed on 4 eyes (4%), to 180° (two quadrants) was performed on 14 eyes (14%), and to 90° (single quadrant) was performed on 5 eyes (5%). No statistically significant difference was observed between the groups in all weeks (Table 4).

**Table 4:** Influence on treatment results of extension of circumferential trabeculotomy, measured through the number of quadrants treated with GATT procedure

	1 (90°)	2 (180°)	3 (270°)	4 (360°)	p-value
Eyes, n	5	14	4	77	
No. of failures	2	3	1	20	0.883
Preoperative IOP	21.20 ± 7.12	26.86 ± 5.76	28.50 ± 9.33	24.53 ± 9.56	0.323
Postoperative IOP					
7 days	20.75 + 8.85	13.00 ± 4.31	11.67 ± 0.58	14.30 ± 7.70	0.255
One month	13.33 + 1.15	13.79 ± 6.78	15.25 ± 4.57	14.86 ± 6.98	0.734
Two months	13.00 + 2.58	11.62 ± 1.56	15.75 ± 4.92	14.16 ± 5.94	0.360
Three months	12.00 + 2.00	11.62 ± 1.56	13.33 ± 1.15	12.76 ± 3.53	0.633
Six months	11.75 + 1.71	12.18 + 1.08	13.67 + 0.58	12.98 + 3.54	0.465
One year	15.00 + 5.66	12.44 + 1.51	13.00 + 1.00	12.48 + 1.94	0.837
Two years	13.00 + 0.00	12.60 ± 1.51	13.00 ± 1.00	12.42+ 1.16	0.886

IOP= intraocular pressure (presented in mmHg).

Values are presented as mean ± standard deviation, unless otherwise noted. ^*^Statistical significance evaluated by Kruskal-Wallis test.

## DISCUSSION

For several years, conventional trabeculectomy with filtering bleb construction has been the gold standard surgical approach for glaucoma. However, complications such as shallow anterior chamber, wound leakage, choroidal effusion, persistent corneal edema, induced astigmatism, encapsulated bleb, bleb-related infection, and bleb leak have been reported to occur in up to 63% of patients in a 5-year follow-up period^(2,16)^. Consequently, there has been an increasing interest in conjunctival-sparing MIGS in the past decade, with an overall goal of reducing failure rates and complications, especially bleb-related complications, and increasing the patients’ quality of life.

Angle-based glaucoma surgery, especially in adult glau coma treatment, has been continuously improving over the past few decades in OAG treatment, with the development of devices and techniques such as Trabectome (NeoMedix Corporation, Tustin, CA)^(17,18)^, canaloplasty^(19,20)^, iStent (Glaukos Corporation, Laguna Hills, CA)^(21,22)^, Kahook Dual Blade (KDB, New World Medical, Rancho Cucamonga, CA), and Hydrus (Ivantis, Inc, Irvine, CA). Although metal trabeculotomies, such as Harms or McPherson, are generally known to have unsatisfactory results in the adult population, GATT has demonstrated as high as 83% success rates^(23)^.

GATT was first described by Grover et al.^(8)^ using both sutures and the iTrack device through a goniotomy and an anterior chamber access. The authors achieved a success rate ranging from 68% to 90%, with minimal complications^(9)^. Furthermore, the mean IOP and number of glaucoma medications were significantly reduced compared with baseline. This study used a thermally blunted 5-0 polypropylene suture and achieved similar success rates as those of previous trabeculotomy studies^(8,14)^. In the current study, we also achieved a reduction in the mean IOP and number of glaucoma medications in all subgroups compared to baseline values, demonstrating the reliability of this technique. To the best of our knowledge, this is the first study conducted in Brazil to address this issue.

When the extent of treatment was evaluated, we did not find different results in terms of mean IOP reduction. Aktas et al. demonstrated that the extent of episcleral venous fluid wave during GATT surgery was a valuable prognostic factor for surgical success, with lesser than 4.5 clock hours of fluid wave being associated with the need for additional treatment^(24)^. Ahuja et al. found that 28% of patients with advanced glaucoma undergoing trabectome surgery required additional intervention compared to only 10% of patients with mild to moderate glaucoma^(25)^. Trabectome surgery, although being Schlemm-canal-based, is not circumferential like GATT. We believe that performing 360° GATT would be related to greater IOP reduction, which is probably true, but we did not find such a result. We could not find a statistically significant difference probably because of the small number of patients in the other groups (i.e., not the 360° GATT group). Future prospective studies with a greater number of patients and comparing GATT with other MIGS such as trabectome, Kahook Dual Blade, and iStent are required to confirm whether the extension of circumferential trabeculotomy is important to achieve better results.

**Figure 2:**
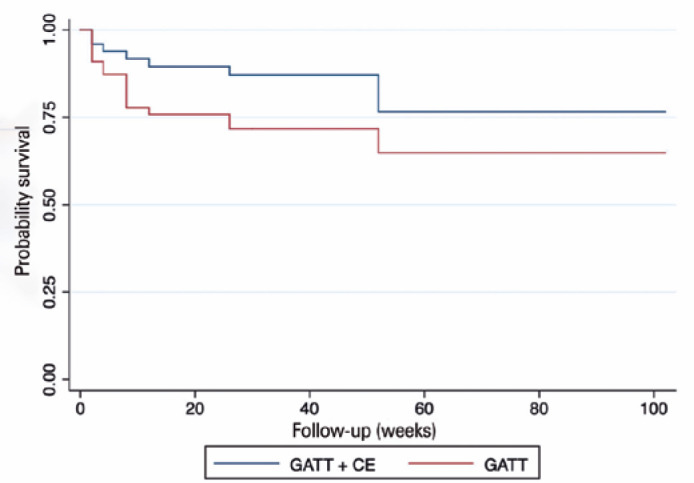
Kaplan-Meier curve demonstrating the probability survival leading to reoperation for poor intraocular pressure control over time separated by study group.

When standalone GATT and GATT associated with CE were compared, no significant success rates were observed. The success rates were 64.9% and 76.6%, respectively, which were not statistically significant (p=0.118) (Figure 2). These results are consistent with previous studies in which concurrent cataract surgery did not have a statistically significant effect on IOP reduction in eyes that underwent GATT procedure^(8,9,14,23)^. The most common postoperative complication was transient hyphema. None of the patients who developed hyphema evolved toward surgical failure, nor any of the complications observed were predictive of further treatment indication. When the complications were minimal and transient, the surgical procedure implies a strong safety profile.

GATT= Gonioscopy-assisted transluminal trabeculotomy; CE= Cataract extraction.

In our study, we observed a high percentage of secondary glaucoma (almost 35%), especially secondary to vitreoretinal surgery and steroid-induced glaucoma. However, both groups apparently had opposite results when subjected to GATT technique. The first group (secondary to vitreoretinal surgery) demonstrated a significant failure, with >65% of patients requiring additional surgery, thus indicating that a tube-shunt or trabeculectomy surgery are probably better options in this subgroup. When excluding patients who underwent vitreoretinal surgery, the failure rate dropped to 17%. It is important to report that these vitreoretinal surgeries were not performed with extensive conjunctival dissection, which could have compromised the patients’ natural outflow pathway. However, 50% of patients (6 patients) received the placement of a retinal buckle as part of their vitreoretinal surgeries, which could have compromised their GATT surgery results. On the other hand, the other group (steroid-induced glaucoma) had 0% failure, suggesting that the GATT technique is especially effective in this subgroup of patients with glaucoma.

There were some limitations of our study. The data represent the surgeon’s learning curve in his first cases. The decision to perform a surgical intervention was purely at the individual surgeon’s discretion, with all the known biases of a not-blinded retrospective study without a controlled group and no randomization. We did not have visual field mean deviation (MD) or other information that could be used to stratify glaucoma into early, moderate, or advanced stages. These limitations could have influenced our results. Grover et al. found that eyes with an MD worse than -15 dB had a greater risk of failure after GATT surgery^(9)^. Moreover, the extension of trabeculotomy was determined only based on intraoperative or anatomical difficulties, and the reduced number of patients who did not undergo 360° GATT could have compromised the statistical analyses of IOP-lowering effect. Furthermore, there was an apparent limitation in the follow-up, as we lost several patients especially at the 2-year follow-up visit and thereafter.

In conclusion, GATT is an apparently safe procedure that emerges as a MIGS, with satisfactory success rates and avoiding the need of bleb construction and preserving the conjunctiva for possible future surgeries. Further prospective studies are warranted to gain a better understanding of the efficacy of GATT and to compare it with standard trabeculectomy and other MIGS procedures.
